# An Apple a Day? The Hypothesis of Cross-Kingdom Gene Regulation by Plant miRNAs in Mammals and Its Controversies

**DOI:** 10.3390/ijms27104220

**Published:** 2026-05-09

**Authors:** Rachele Matsagani, Paola Monti, Federica Rota, Eva Dariol, Elia Mario Biganzoli, Valentina Bollati

**Affiliations:** 1 ‘LETE’ Laboratory of Environmental and Translational Epigenetics, Department of Biomedical and Clinical Sciences, University of Milan, 20157 Milan, Italy; rachele.matsagani@unimi.it (R.M.); eva.dariol@unimi.it (E.D.); elia.biganzoli@unimi.it (E.M.B.); 2 ‘INES’ Initiative of Epigenetics for Smiles, University of Milan, 20157 Milan, Italy; 3 Department of Clinical Sciences and Community Health, “Dipartimento di Eccellenza 2023–2027”, University of Milan, 20122 Milan, Italy; paola.monti@unimi.it (P.M.); federica.rota@unimi.it (F.R.)

**Keywords:** microRNA, plant, cross-kingdom regulation, gene expression

## Abstract

MicroRNAs (miRNAs) are small non-coding RNAs that negatively regulate gene expression at the post-transcriptional level. In plants, miRNAs are involved in environmental responses and can be transferred to other species to mediate cross-kingdom regulation of gene expression. This mechanism has recently been proposed in mammals, yet evidence remains scarce and inconsistent. Multiple studies have shown that a fraction of plant-derived miRNAs (pmiRNAs) present in food resist processing, cooking, and digestion. Evidence suggests that dietary-derived pmiRNAs might be absorbed in the gastrointestinal tract and enter circulation, predominantly packaged in extracellular vesicles, and reach different tissues, where they might exert cross-kingdom gene expression regulation. Nonetheless, several attempts to reproduce or confirm the above results have failed, raising questions about the reliability of studies supporting the hypothesis of cross-kingdom gene regulation by pmiRNAs. In this review, we recapitulate the state-of-the-art knowledge in the field, addressing both supporting and opposing evidence, as well as the main analytical challenges that need to be taken into consideration, in an effort to provide a comprehensive framework on the controversial evidence collected so far and support the use of best practices for future research.

## 1. Introduction

MicroRNAs (miRNAs) are highly conserved, 18–24 nucleotide-long single-stranded non-coding RNAs found in a variety of organisms, including plants and animals. By targeting complementary sequences, they negatively regulate gene expression at the post-transcriptional level, either through target mRNA cleavage, mostly followed by degradation, or translational repression [1].

MiRNAs are identified with numbering (e.g., miR-121) with a prefix indicating the organism of origin (*Homo sapiens*, hsa) and numbered or lettered suffixes indicating identical miRNA sequences originating from distinct precursors (hsa-miR-121-1 and hsa-miR-121-2) or closely related mature miRNAs (hsa-miR-121-1a and hsa-miR-121-1b), respectively [2].

Biogenesis of both plant and animal miRNAs initiates within the nucleus with the transcription of a primary miRNA (pri-miRNA) with a stem-loop structure [3,4]. In animals, pri-miRNAs are cleaved in the nucleus by Drosha to yield pre-miRNAs, which are transported in the cytoplasm and further processed by Dicer to form the mature miRNA/miRNA* duplexes [3,5,6,7,8]. In plants, pri-miRNAs are cleaved in the nucleus in two steps by Dicer-like 1 (DCL1) to form the mature miRNA/miRNA* duplexes [9]. After methylation at the 3′-terminal by HEN1, duplexes are exported in the cytoplasm [10,11]. For miRNAs to be functional, it is necessary that one strand of the duplex, the ‘guide strand’, is incorporated in the RNA-induced silencing complex (RISC) [12,13,14]. Once loaded in the RISC, miRNAs silence their target mRNA either through mRNA cleavage or translational repression, which is determined by the extent of sequence complementarity between the miRNA and its target [15]. In plants, miRNA’s high complementarity with the target primarily promotes target cleavage by RISC, although translational inhibition has also been described [16]. In contrast, in animals, miRNA-target complementarity is imperfect, resulting in translational inhibition [17]. Interestingly, both in plants and animals, miRNAs can be transported within extracellular vesicles (EVs) to distant cells, and participate in intercellular communication [18].

Both in plants and animals, miRNAs generally target important transcription factor families, such as the GROWTH REGULATING FACTORs (GRFs) or the SQUAMOSA PROMOTER BINDING PROTEIN-LIKE (SPL), hence regulating a variety of processes. Indeed, plant miRNAs (pmiRNAs) are key regulators of plant growth, development, and phenotypic plasticity triggered by various environmental stimuli (e.g., light, temperature, and nutrients) (extensively reviewed in [19,20]). They also mediate communication among plants and interactions with symbionts and parasites, thereby contributing to ecological adaptation. It is well-known that during infection by pathogens, pmiRNAs not only mediate mechanisms of immunity, but can also be packed into EVs and exported into pathogens to repress virulence and disease-related proteins [20]. This mechanism illustrates the evolutionary versatility of miRNAs as agents of inter-species communication. Beyond plant-pathogen interplay, the hypothesis that pmiRNAs could mediate cross-kingdom regulation in mammals has increasingly drawn attention [21,22]. This assertion is corroborated by the fact that plants and mammals share many common biological processes, including miRNA biogenesis and mechanism of action, although some differences exist; indeed, some pmiRNAs have been found to possess sequence homology and suggested to exert functions similar to those of mammalian miRNAs. By using bioinformatic approaches, Pirrò et al. identified 9 miRNAs with 57% to 78% sequence homology with human miRNAs. Among those pmiRNAs, mol-miR-168a, which shares 57% homology with hsa-miR-579, was able to downregulate its target gene SIRT-1 in vitro [23]. Similarly, the 21-nt aba-miR-9497 from the medicinal plant *Atropa belladonna* and the 21-nt human brain hsa-miR-378 share 76% sequence homology and were both shown to target, in vitro, the human zinc-finger transcription factor ZNF-691 mRNA [24].

Since the seminal report by L. Zhang et al. suggesting dietary transfer of rice-derived miR-168a into mouse liver [21], interest in this phenomenon has grown rapidly. However, the scientific community remains deeply divided. While some studies claim to detect pmiRNAs in mammalian tissues and biofluids [25,26,27,28,29,30,31,32,33,34,35,36,37], others argue that such findings are likely artifacts or contamination [38,39,40,41,42,43,44,45,46,47,48,49,50,51,52,53]. Moreover, a key unresolved question concerns the biological plausibility of this process: how can dietary pmiRNAs survive GI digestion, cross epithelial barriers, and specifically recognize mammalian mRNA targets [54,55,56,57,58,59,60]?

Although evidence remains limited and controversial, understanding whether pmiRNAs can genuinely exert cross-kingdom regulatory effects in mammals is of considerable conceptual and practical relevance, with implications for medicine, nutrition, and ecology. In this review, we critically synthesize current knowledge by contrasting supporting and opposing evidence from studies published after 2012. We also discuss the main methodological and analytical challenges that may account for inconsistent findings and propose best practice guidelines for future research.

## 2. Evidence Supporting the Hypothesis of Cross-Kingdom Regulation by Dietary pmiRNAs in Mammals

Following the initial report by L. Zhang et al. suggesting that plant miRNAs could cross the GI barrier and modulate mammalian gene expression, several groups expanded on this finding by investigating the presence and potential activity of dietary pmiRNAs in mammalian systems [21]. L. Zhang and colleagues detected plant miR-156a, miR-168a, and miR-166a in human and animal serum, in serum-derived EVs, and in multiple mouse tissues following rice feeding [21]. They further demonstrated that miR-168a could bind to the human and mouse low-density lipoprotein receptor adapter protein 1 (LDLRAP1) mRNA, leading to reduced hepatic expression of LDLRAP1 and a consequent decrease in LDL clearance from plasma [21].

The same year, K. Wang et al., by adopting a stringent bioinformatics approach, showed that a significant fraction of RNAs in human plasma were of exogenous origin, including food-derived sequences, namely miR-168 from different commonly consumed cereals [25]. The following year, the same group identified miR-166 and miR-168 from corn and rice after sequencing mouse plasma RNA samples [26]. The presence of these miRNAs was attributed to diet, since mice were kept in a controlled environment, supporting the evidence of pmiRNA transfer to mammals by oral intake [21,25,26].

With the growing interest in the field, subsequent research efforts have primarily focused on three critical aspects of this hypothesis: (1) the stability of pmiRNAs during food processing and digestion, (2) the mechanisms of pmiRNA absorption and transport across the GI barrier, and (3) their potential cross-kingdom regulatory effects in mammalian cells and tissues.

In the following subsections, we summarize and critically appraise the main findings concerning each of these key aspects.

### 2.1. Studies on pmiRNA Stability

The stability of pmiRNAs before ingestion and during early digestion is a fundamental prerequisite for their cross-kingdom effects. For this reason, multiple studies have tried to address this issue by analyzing pmiRNA recovery after storage, processing, and cooking of plant-derived food [32,58,61,62,63]. Philip et al. were the first in 2015 to investigate this aspect by examining the stability of selected abundantly present pmiRNAs during storage, soaking, and cooking of rice and soybean, as well as during in vitro mastication and early digestion [55]. Notably, they observed that miR-166, miR-167, and miR-168 were abundant in food after processing and their levels remained constant during simulated digestion, suggesting that pmiRNAs could survive degradation during food preparation, as well as in the GI tract [55]. Similarly, miRNA levels in raw broccoli heads, sprouts, and juice were found to resist different heat-processing methods and in vitro digestion, with recovery levels oscillating between 0.1 and 10% if compared to the unprocessed counterparts [57].

Subsequent research focused on the biochemical determinants of pmiRNA stability. X. Wang et al. observed variable but reproducible degradation profiles among different pmiRNAs in human saliva and in gastric and intestinal fluids [56,60]. In human saliva, pmiRNAs were shown to undergo an initial extensive degradation by salivary RNase, after which their concentration remained constant. Intriguingly, the stability of pmiRNAs in saliva was increased by the presence of 3′ end 2′-O-methylation or by the presence of the main food components (starch, protein, and lipid) [60]. In recent decades, it has become increasingly evident that the nutritional properties of foods are influenced by the structural organization and interactions of food constituents, a phenomenon termed the food matrix effect [64]. During mastication and digestion, food components form micro- and nanostructures of different types (e.g., lipid emulsions, protein- or starch-based gels), which can influence the stability and bioavailability of various compounds present in food. Such effects arise from alterations in the thermodynamic and kinetic properties of the chemical reactions that govern digestion and absorption processes. For example, partitioning of reactants between distinct phases, such as in emulsions, affects their local concentrations and interaction probabilities. The presence of structural barriers, macromolecular crowding, or increased viscosity, as observed in gel-like systems, reduces molecular diffusion and, consequently, collision events [65]. Within this framework, food matrices might increase pmiRNA stability by encapsulating them in macro- and nanostructures, thereby limiting their exposure to RNase activity. In line with this, EVs from plant foods enhanced the resistance of their pmiRNA cargo, especially in the presence of the food matrix [60]. Confirming this result, another study has demonstrated that EVs protect miRNAs from RNase degradation [66]. Furthermore, the survival rate of all pmiRNAs in saliva increased dramatically by decreasing their initial concentrations, supporting previous findings stating that the efficiency of enzymatic digestion on both DNA and RNA is reduced by the limited copy number of the target molecule [60,67,68]. Instead, the stability of pmiRNAs in gastric juice was not influenced by the presence of 3′ end methylation but was increased by the presence of food components. Conversely, in intestinal fluid, a strong degradation of all pmiRNAs was observed within 2 h, which was effectively reduced by 2′-O-methylation, but not by the presence of the food matrix. Moreover, the most resistant molecules, namely miR-159, miR-168, miR-160, and were shown to exhibit stem-loop secondary structures and self-pairing at 3′ termini, features associated with increased stability. In contrast, for miR-157 and miR-894, folding into self-pairing structures was predicted to be unlikely under physiological conditions, which could explain their substantial degradation in gastric and intestinal fluid. Interestingly, miR-172, despite exhibiting a stem-loop structure, was largely degraded in intestinal fluid, possibly due to its free 3′ terminus, which could be recognized by RNase [56]. These results suggest that 3′ end protection by secondary structures and methylation can hamper RNase degradation in saliva and intestinal fluid, but not in gastric juice, in which the main digestive enzyme is pepsin. On the other hand, the protective role of food constituents is lost in intestinal fluid, where pmiRNA-containing micro- and nanostructures are degraded during digestion.

Overall, evidence shows that processing and cooking, as well as early digestion of plant-derived foods, contribute to substantial degradation of pmiRNAs. However, a fraction of pmiRNAs resists these adverse conditions, owing to intrinsic factors, including 3′ terminal 2′-O-methylation modification and a secondary structure featuring stem-loops and self-base pairing at the 3′ end, as well as protection provided by extrinsic factors, including EVs and food components. Consequently, non-degraded and non-digested pmiRNAs could be absorbed in the GI tract and distribute in the organism through circulation. A comprehensive summary of the studies discussed in this section is provided in Table 1.

### 2.2. Studies on the Kinetics and Mechanism of pmiRNA Absorption

In parallel to studies on pmiRNA stability, several groups examined the kinetics and mechanisms of pmiRNA absorption in mammals [28,29,32,35]. Multiple reports detected pmiRNAs in plasma or serum following oral intake of vegetables, suggesting that—similarly to other food-borne miRNAs, such as those contained in cow milk—certain pmiRNAs may cross the GI barrier and enter circulation [45,69]. In 2015, Liang and colleagues identified 6 pmiRNAs showing progressively increasing concentrations (in the femtomolar range) in plasma of volunteers after drinking watermelon juice or eating mixed fruits following overnight fasting. Concentrations peaked between 1 and 3 h after ingestion, with estimated absorption efficiencies of 0.04–1.31% relative to initial dietary levels. Among these, miR-156a and miR-172a had a concentration range similar to that of endogenous miRNAs [29]. Similarly, lettuce ingestion following overnight fasting led to a gradual increase in serum miR-156a with peak levels reached 1 to 3 h after feeding, although the absorption efficiency varied between individuals [35]. Feeding mice with total RNAs extracted from cabbage resulted in transient detection of miR-172 and miR-824 in serum, stomach, intestine, liver, spleen, kidney, and feces within 2 to 4 h after ingestion, persisting up to 3 days [28]. In pigs, Luo et al. reported that a single corn meal induced a rise in circulating pmiRNA within 6 to 12 h, while repeated feeding for 7 days yielded a stable concentration of pmiRNAs in serum [32].

Although these findings suggest limited but measurable uptake of dietary pmiRNAs, the underlying mechanisms remain incompletely understood. Evidence indicates that intestinal epithelial cells can internalize pmiRNAs through clathrin- and caveolin-dependent endocytosis, as well as receptor-mediated processes [56]. Chen et al. identified the mammalian RNA transporter SID-1 transmembrane family member 1 (SIDT1) expressed on gastric pit cells as a mediator of dietary miRNA uptake, showing that an acidic environment enhances SIDT1-dependent internalization in primary gastric epithelial cells [59]. The contribution of plant-derived EVs in pmiRNA uptake by intestinal cells was also demonstrated by independent groups. Ju and colleagues showed that EVs from grape could reach the murine intestine after oral administration and be taken up by intestinal stem cells via macropinocytosis [70]. Further in vitro studies showed that pmiRNA-containing ginger EVs were internalized by Caco-2 enterocyte-like cells via caveolin-mediated endocytosis and micropinocytosis and displayed perinuclear localization [71]. Uptake of miRNAs transported within broccoli, apple, pomegranate, and orange EVs by Caco-2 cells was also shown in a later study [66]. Similarly, in rat enterocytes (IEC6), time-dependent uptake and perinuclear localization of EVs from grapefruit juice were observed, accompanied by increased levels of their cargo miR-168a [72]. Permeability of pmiRNAs was also investigated via alteration of mice GI integrity by cisplatin treatment, which resulted in enhanced bioavailability of miR-168a in mice fed with honeysuckle decoction supplemented with miR-168a [73]. However, in a later study by the same group, alteration of mice GI permeability by treatment with either aspirin or anti-CD3 antibody did not change pmiRNA bioavailability, indicating that more research on the mechanisms of free or encapsulated pmiRNA absorption by intestinal cells is needed [74].

In summary, current evidence suggests that dietary-derived pmiRNAs might be absorbed in the GI tract by either transporter or receptor-mediated mechanisms, as well as endocytosis, resulting in a gradual increase in circulating pmiRNAs levels reaching peak concentrations a few hours after food intake. These findings further support the hypothesis of cross-kingdom transfer of miRNAs from plants to mammals. However, additional research is needed to better understand the kinetics and mechanisms of pmiRNA absorption as well as the role of plant and/or mammalian EVs in this process.

### 2.3. Studies on pmiRNA Cross-Kingdom Activity

Research on pmiRNA transfer in mammals has been accompanied by studies exploring their potential cross-kingdom regulatory activity. A first line of research has focused on in silico prediction of pmiRNA target genes in several mammalian species [23,27,33,36,63,75,76]. In one large-scale analysis of 410 publicly available plasma small RNA-sequencing (sRNA-seq) datasets, the two most abundant pmiRNAs (miR-2910 and miR-2916) were found to share seed sequences with human miRNAs (miR-4259, miR-4715-5p, and miR-4652-5p), and their targets were predicted to be involved in basic cell metabolism processes, such as regulation of transcription and RNA splicing [31]. Besides, Olmi et al., by adopting a stringent bioinformatics approach for the identification of pmiRNAs in previously published plasma sRNA-seq data, showed that the mammalian molecular pathways putatively regulated by the 350 detected pmiRNAs were implicated in neurogenesis and nervous system development [37].

A second major research direction has examined plant and mammalian EVs as carriers of pmiRNAs, suggesting an additional mean by which pmiRNAs might be delivered and modulate gene expression in mammalian cells. EVs derived from different plants have recently attracted attention for their potential as carriers of plant-specific molecules with beneficial effects on human health [77]. Although most research has focused on bioactive compounds (e.g., polyphenols, vitamins), a few studies have investigated the role of pmiRNAs in plant-derived EVs in the molecular changes they elicit. In Caco-2 cells, uptake of pmiRNA-containing ginger EVs, but not miRNA-depleted EVs, inhibited the LPS-induced expression of NF-κB and pro-inflammatory cytokines [71].

Interestingly, the interaction of plant EV-contained pmiRNAs with the gut microbiota has also been investigated, providing an additional potential means by which these exogenous miRNAs may influence the host. Few independent studies have reported the uptake of EVs derived from various plants (ginger, turmeric, garlic, grapefruit, mulberry bark, and tartary buckwheat) by different bacterial species [78,79,80]. Oral administration of ginger-derived EVs could modulate the gut microbiota, improve barrier function, and reduce bacterial translocation into peripheral blood in a mouse model of acute colitis. These effects were associated with modulation of the expression of the monooxygenase ycnE and the pilus protein SpaC by ginger EV miR-7267-3p and miR-167a, respectively, in the probiotic bacterium *L. rhamnosus* [81]. In the same mouse model, orally administered garlic-derived EVs mitigated colon lesions and epithelial barrier impairment, effects that were accompanied by changes in fecal microbiota richness and diversity, particularly involving the gut-health-promoting genus of *Bacteroides*. In vitro, garlic EVs could enhance *B. thetaiotaomicron* proliferation, which was attributed, at least in part, to the delivery of the abundant cargo peu-miR-2916-p3 [82]. Two independent groups showed that dietary supplementation with free pmiRNAs enhanced bacterial abundance and diversity in the mouse intestine, further supporting the beneficial role of pmiRNAs, either free or EV-transported, in gut homeostasis preservation [83,84]. Remarkably, a recent study detecting pmiRNAs in feces but not in blood from human volunteers, proposed that the primary biological activity of dietary pmiRNAs might be restricted to the intestinal lumen, affecting microbial communities rather than host tissues [64].

Beyond plant-derived EV transport, several studies have identified an enrichment of pmiRNAs in host EVs [21,29,30,32,35,75,85,86]. Packaging and secretion of pmiRNAs within EVs was observed following internalization by gastric epithelial cells in vitro [59]. Intriguingly, multiple pmiRNA species were detected in publicly available sRNA-seq data from human and porcine breast milk EVs. The top predicted targets for the 5 most abundant pmiRNAs identified in human milk EVs (namely, ath-miR-319b, ctr-miR-167, ath-miR-166a, zma-miR-156a, and osa-miR-444b.2) were associated with immune system function, hormone response, and transcription regulation [27]. In a subsequent investigation by the same group, target prediction was performed for 5 pmiRNAs detected in breast milk and corresponding EVs from healthy donors. Functional enrichment analysis indicated that these pmiRNAs preferentially target mRNAs involved in protein localization, enzyme binding, and intracellular signal transduction, suggesting a potential regulatory role in neonatal cellular homeostasis [34]. Supporting this evidence, 37 mature bamboo pmiRNAs were also identified in sRNA-seq data of EVs from giant panda breast milk [27,33]. Interestingly, the abundance of milk EVs pmiRNA changed in accordance with different lactation periods, which could be attributed to the different food intake by giant panda mothers after delivery. Moreover, functional enrichment analyses of the putative target genes indicated that the top pmiRNAs identified (namely, dla-miR-156e-5p, dla-miR-168a-5p, dla-miR-319a-3p, dla-miR-535-5p, dla-miR-156h-5p, dla-miR-1310-5p, dla-miR-1310-3p, dla-miR-1311-3p, dla-miR-2916-5p, and dla-miR-2916-3p) were primarily involved in basic cell metabolism processes, as well as neurodevelopmental processes, such as synapse organization, neuron migration and axon guidance [33]. These studies, in addition to supporting the hypothesis of pmiRNA transfer in mammalian organisms through EVs, suggest the potential role of pmiRNA-containing EVs in post-natal neurodevelopment, a mechanism already known to be true for mammalian miRNAs present in breast milk [87].

Finally, many research groups have also investigated the activity of pmiRNAs in vitro and in vivo, to unravel their function as well as therapeutic potential. After observing decreased serum miR-159 levels in breast cancer (BC) patients compared to healthy donors, which were inversely correlated with BC incidence and progression, Chin and colleagues demonstrated for the first time in 2016 that orally administered miR-159 could inhibit cancer growth in vivo. In vitro, miR-159 downregulated TCF7, a transcription factor of the Wnt signaling pathway that is upregulated in BC and promotes tumor growth [30]. Supporting pmiRNA anti-proliferative effects by targeting the Wnt signaling pathway, in enterocytes, the highly conserved miR-167e-5p downregulated β-catenin, while miR-156—a pmiRNA abundant in green vegetables—inhibited Wnt10b expression [88,89]. Similarly, miR-156a was detected at high concentrations in serum of healthy individuals, whereas it exhibited lower levels in serum and blood vessels of patients with cardiovascular disease (CVD). In vitro, miR-156a targeted junction adhesion molecule-A (JAM-A), which is upregulated in atherosclerotic lesions, and reduced inflammation-induced monocyte adhesion, suggesting a novel mechanism through which leafy greens could provide vasoprotection [35]. Targeting of JAM-A by miR-156a was also demonstrated in vitro in the context of nasopharyngeal cancer, in which it repressed epithelial–mesenchymal transition [90]. Other studies have revealed the potential therapeutic capabilities of pmiRNAs in several pathologies, including inflammatory conditions, cancer, and metabolic diseases [58,71,91,92,93,94,95,96,97,98,99,100,101].

Several studies have shown that orally ingested pmiRNAs can influence gut homeostasis and modulate bacterial abundance and diversity, and can enter circulation, mostly within EVs, and reach mammalian tissues where they may affect gene expression. Nevertheless, these findings remain preliminary and context-dependent. Moreover, only a few studies have validated pmiRNA-mammalian mRNA interaction through functional assays (e.g., dual-luciferase reporter assays) [21,24,30,32,35,84,88,89,90,95,98,100]. Notably, the detection of EV-encapsulated pmiRNAs in breast milk further suggests a potential, though yet unproven, mechanism of intergenerational transfer influencing post-natal development.

## 3. Evidence Opposing the Hypothesis of Cross-Kingdom Regulation by Dietary pmiRNAs in Mammals

The pioneering study by L. Zhang et al. (2012) sparked an intense debate, and since then, many groups have argued that evidence for exogenous miRNAs’ presence and function in mammals may largely reflect contamination or technical artifacts [43,51,52].

### 3.1. Contamination and Sequencing Artifacts

Y. Zhang et al. were the first to challenge the idea that dietary uptake of pmiRNAs is a general feature of animals [21,38].

By re-analyzing 83 publicly available sRNA datasets from multiple species (mammals, chicken, and insects) using different sampling techniques and experimental and analytical methodologies, the authors detected several pmiRNAs, mostly miR-168, but in extremely low abundance. Moreover, nearly all miR-168 sequences were of monocot origin, even in insects fed exclusively on dicot plants.

These inconsistencies, together with shared miRNA sequences among samples from different organisms processed and sequenced in the same laboratories, led the authors to attribute the findings to cross-sample contamination during multiplexing rather than true biological transfer [38]. Several independent analyses later supported this view [44,50,53]. Tosar et al. compared two datasets from L. Zhang et al. and found a striking correlation in pmiRNA profiles between unrelated experiments, consistent with a batch-specific contamination effect [43].

In addition, the same group also pointed out that the mouse feeding regimen used by L. Zhang et al. would correspond to an unrealistic human intake of 33 kg of rice per day [21,42].

### 3.2. Lack of Reproducibility and Physiological Plausibility

Dickinson et al. failed to reproduce L. Zhang and colleagues’ findings under controlled feeding conditions. Although mice fed rice-based chow showed increased LDL levels, they exhibited no reduction in hepatic LDLRAP1, contradicting the proposed mechanism [21,39]. The LDL increase was instead attributed to metabolic compensation for low dietary cholesterol [39].

Similarly, the robustness of later studies was questioned, highlighting similar methodological weaknesses, including the detection of pmiRNAs absent in the plant species eaten during the experiment, high cross-sample correlations indicative of sequencer carry-over and use of non-physiological amounts of consumed plants [46,47].

A comprehensive meta-analysis of 824 sequencing datasets from human tissues and body fluids provided further evidence for contamination. Exogenous miRNAs were not enriched in tissues with dietary exposure, most reads derived from non-food organisms (e.g., rodents and insects), and identical pmiRNA profiles were shared among samples from the same study (batch effect) [49].

### 3.3. Misinterpretation of miR-2911

Another line of critique concerns the frequently cited miR-2911, an exceptionally stable sRNA due to its high GC content [102,103,104,105,106,107]. Recent analyses indicate that miR-2911 originates from rRNA fragmentation rather than canonical miRNA processing [108,109,110]. It lacks association with EVs or AGO proteins composing the RNA-induced silencing complex (RISC) and does not function through standard RNA-silencing pathways. Moreover, its high GC content likely promotes aggregation and artifactual stability, complicating detection and mapping [103,104,105,106,107,108,109,110,111].

### 3.4. Negative Feeding Studies

Finally, several independent feeding studies in mammals, including humans, failed to detect any pmiRNA in plasma or tissues. When weak (late) amplification signals were obtained, they were inconsistent across replicates, reflecting background noise rather than true detection [40,41,45,48].

In summary, converging evidence from re-analyses, meta-analyses, and negative feeding studies strongly suggests that previously reported dietary pmiRNAs in mammals are more plausibly explained by contamination, sequencing artifacts, or misinterpretation of non-miRNA fragments. These findings do not fully exclude cross-kingdom transfer, but they emphasize that robust validation, physiological dosing, and standardized negative controls are essential before accepting dietary pmiRNAs as functional regulators in mammals.

## 4. Technical Challenges to the Study of pmiRNAs in Mammals

As the debate over the validity of the hypothesis of cross-kingdom regulation of gene expression by pmiRNAs in mammals evolved, the challenges and limitations posed by the currently available methodologies also became a topic of discussion. In this section, we discuss the main pitfalls and caveats of the most widely employed techniques for pmiRNA extraction and quantification, with a focus on sRNA-seq and quantitative reverse transcriptase-PCR (qRT-PCR), both of which are prone to bias and false positives. We also emphasize that special care should be taken to minimize all potential sources of contamination during library preparation and sequencing, as well as the qRT-PCR protocol.

### 4.1. PmiRNA Extraction

RNA extraction is a critical step in the pmiRNA analysis pipeline, as it can strongly influence miRNA content and diversity. A 2015 study showed that phenol/chloroform-based approaches outperformed column-based methods (mirVana™, Thermo Fisher Scientific Inc., Waltham, MA, USA) in terms of reproducibility and sensitivity for detecting low-abundance pmiRNAs [29]. Although similar evaluations have been conducted on human miRNAs [111], no additional comparative reports have been published since, highlighting the need to better understand how current extraction methods affect downstream pmiRNA detection. Moreover, several commercially available miRNA extraction kits have been shown to introduce sRNA contaminants, whose proportion is inversely correlated with the input amount of biological material. Careful selection of the extraction method is therefore required, taking into account yield, reproducibility, and potential contamination. Additionally, rigorous control of sample handling procedures and RNA isolation steps is essential to minimize contaminants, particularly when assessing exogenous RNAs, regardless of the extraction method [112].

When pmiRNAs within EVs are investigated, the pre-processing steps of EV isolation prior to miRNA extraction should be performed following the MISEV recommendations and accurately reported. Indeed, the lack of a standardized method for EV isolation results in different enrichment of EVs over other non-vesicular particles (e.g., protein and ribonucleoprotein (RNP) aggregates) that may co-isolate with them, thereby influencing the outcome, and contributing to the contradictory results obtained so far [113].

### 4.2. PmiRNA Analysis by sRNA-Sequencing

Following RNA extraction, the typical sRNA library preparation workflow involves adapter ligation, RT, and PCR amplification. It has long been known that 3′ terminal 2′-O-methylation of pmiRNAs interferes with poly(A) and poly(U) tailing and with T4 RNA ligase-mediated adapter ligation. The efficiency of those reactions depends on enzyme concentration, buffer composition, incubation conditions, and RNA base composition and structure [114]. Consequently, under-representation of plant sequences often occurs in mixed plant-animal cDNA libraries, which might explain the low abundance of pmiRNAs in many publicly available sequencing datasets and, at least in part, inconsistencies among different studies [114,115,116].

For example, in the study by Dickinson et al., only ~1000 reads per million corresponded to rice miRNAs in chow containing 75% rice, inconsistent with previous estimates that miRNAs in rice represent ~10% of total reads [117].

For more accurate comparison of plant and animal miRNAs, optimization of reaction parameters is recommended, including extended incubation times, increased enzyme concentration, and the use of molecular crowding agents such as polyethylene glycol (PEG) [114].

In addition, miRNAs can be treated with sodium periodate prior to sequencing. Sodium periodate oxidizes free 2′ and 3′ hydroxyls of mammalian and non-mature plant miRNAs, but not methylated mature pmiRNAs, allowing only the latter to be ligated to adapters, and therefore sequenced [118]. This treatment helps confirm the plant origin of identified miRNAs, but requires large input RNA amounts and introduces additional processing steps [117]. Only a few studies have introduced periodate oxidation in their protocols [22,31,33,34,37,44,117,119].

Furthermore, while adapter ligation contributes the most to bias during library preparation, the PCR step can also distort quantification, due to varying amplification efficiencies across molecules of different lengths and secondary structures [120,121]. Amplification steps are also a well-known major source of contamination [44]. Sequencing depth must likewise be chosen carefully, as it affects both the number of detected miRNAs and the presence of contaminants [45,112].

Analysis of sRNA-seq data is also challenging and requires the development of a stringent, reliable and reproducible bioinformatics protocol for identifying putative pmiRNAs without ambiguity or false positives. Although some studies have attempted it, evidence in this regard remains limited [27,36,37]. Finally, comparison of libraries prepared in the same time frame and using the same equipment should be carried out when analysing sequencing data from multiple sources (e.g., online, publicly available repositories) [44].

### 4.3. PmiRNA Analysis by qRT-PCR

In qRT-PCR assays, the poly(A)-tailing approach results in a reduced amplification efficiency of methylated miRNAs compared to non-methylated ones, due to the interference exerted by the 3′ terminal 2′-O-methylation, as described above [116]. Stem-loop qRT-PCR should therefore be used, as it provides specific, accurate, and reliable detection of pmiRNAs [117,122]. The stem-loop qRT-PCR method employs stem-loop RT primers with a short single-stranded region complementary to the miRNA 3′ end, a double-stranded stem, and a loop containing the universal primer-binding sequence. Advantages include reduced non-specific hybridization to miRNA precursors or longer RNAs and better discrimination among closely related miRNA family members [118].

Stem-loop qRT-PCR is also preferable to sequencing for the detection and quantification of pmiRNAs, especially when present at low abundance in the analyzed samples. Nevertheless, stem-loop qRT-PCR does not discriminate between unmethylated and methylated 3′ ends, therefore between immature and mature pmiRNAs, respectively. To overcome this limitation, alkaline β-elimination following sodium periodate treatment has been used, removing unmethylated 3′ terminal nucleotides, therefore allowing the amplification of mature pmiRNAs only, as they are protected by the 3′ end 2′-O-methylation [83,118]. Also in this case, large input RNA amounts are required, and additional processing steps are introduced in the protocol [116].

Inclusion of proper controls (e.g., no-template controls, internal controls, and spike-in controls) is fundamental for monitoring the reaction efficiency (e.g., RT, periodate oxidation, and β-elimination, etc.), detecting contaminants, and ensuring accurate quantification of pmiRNAs in samples by qRT-PCR [30,113,123]. However, no universally accepted reference controls exist for circulating miRNAs. Very few studies on pmiRNA transfer in mammals have used internal and/or spike-in controls. Endogenous miRNAs used include miR-16, miR-21, miR-25, miR-92a, miR-103a, miR-122, miR-141, miR-196, and let-7d/g/i, while various synthetic methylated and unmethylated miRNAs, selected among those not present in the examined material, have been used as spike-in controls [29,39,41,61,63,116,118].

Spike-ins should be added at concentrations comparable to those of pmiRNAs to enable accurate normalization [99]. Moreover, when performing qRT-PCR, the use of specific standard curves for each pmiRNA is crucial for absolute quantification and for determining assay efficiency, linear dynamic range, and reproducibility of the assay [21].

A comprehensive summary of the pitfalls and biases encountered at the different steps of the procedures used in studies on pmiRNA cross-kingdom gene regulation, discussed in this and previous sections, is provided in Table 2.

## 5. Discussion

**Figure 1:**
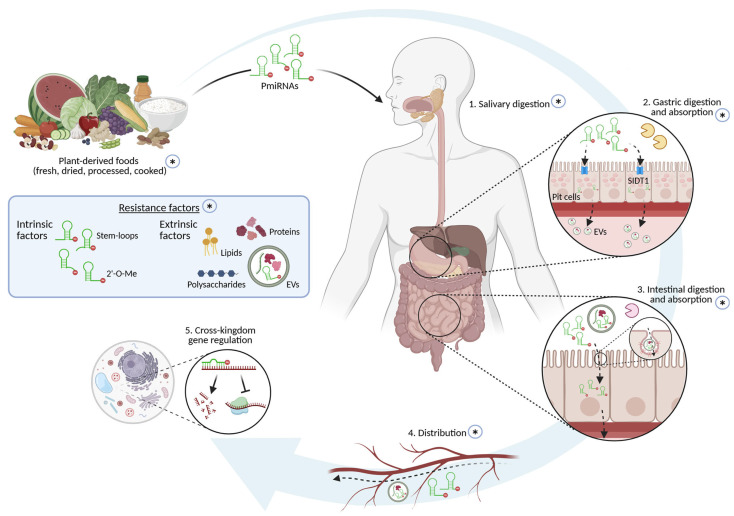
PmiRNA journey from plant-derived foods to human recipient cells. (Created in BioRender. Matsagani, R. (2026) https://BioRender.com/t2th32n).

Since the first report in 2012 suggesting dietary uptake of pmiRNAs and cross-kingdom regulation in mammals, multiple studies have provided evidence supporting the hypothesis that pmiRNAs can withstand the harsh conditions posed by food processing, mastication, and digestion. Once in the mammalian GI tract, pmiRNAs might be internalized by epithelial cells, transported to the bloodstream, predominantly packaged in EVs, and delivered to different tissues, where they might exert cross-kingdom regulation of gene expression and potentially mitigate pathological processes (Figure 1). Moreover, pmiRNAs have been detected in milk, suggesting a role in post-natal development.

Recent findings have expanded this paradigm by suggesting an interaction between dietary pmiRNAs and the gut microbiota, providing an additional and biologically plausible route through which plant-derived miRNAs could influence mammalian physiology. In this regard, pmiRNAs were shown to modulate bacterial abundance/diversity and metabolite production, as well as reduce bacterial translocation into circulation, thus contributing to the maintenance of gut homeostasis [64,82,83,85]. Interestingly, a recent study detecting pmiRNAs in feces but not in blood from human volunteers, suggested that the biological activity of dietary pmiRNAs might be restricted to the intestinal lumen, affecting the microbiota rather than host physiology [63]. Given that miRNA–bacteria interactions are well established in plants, the notion that pmiRNAs could shape the mammalian microbiota through similar mechanisms is conceptually sound, though it remains experimentally underexplored.

Beyond ingestion, an unexpected route of horizontal pmiRNA transfer was proposed by Koupenova et al., who identified three pine-pollen miRNAs (pde-miR-946, pta-miR-1310, and pta-miR-948) in publicly available sequencing data from plasma-derived RNA samples and hypothesized that inhalation might represent another mechanism of exposure [37]. Although intriguing, these results require further validation to rule out environmental contamination and confirm biological relevance.

Despite mounting circumstantial evidence, two major issues limit acceptance of the cross-kingdom hypothesis: biological plausibility and technical robustness. For pmiRNAs to regulate mammalian genes, they must (i) survive digestion, (ii) be absorbed and distributed systemically in biologically effective concentrations, and (iii) interact with target mRNAs with sufficient stoichiometric strength. Current quantitative estimates suggest that circulating pmiRNAs are typically found at femtomolar levels, several orders of magnitude lower than endogenous miRNAs known to exert post-transcriptional regulation. Considering typical miRNA–mRNA binding affinities and turnover rates, such concentrations are unlikely to produce measurable gene-silencing effects under physiological conditions [54,124,125,126].

However, little is known about the mechanism of pmiRNA transfer in circulation and cellular uptake [59,72]. Moreover, key parameters such as miRNA copy number, target availability, compartmental localization, and competition with endogenous miRNAs or long non-coding RNAs remain largely undefined.

Regarding the presence of food-borne pmiRNAs in breast milk, additional steps, namely distribution in the mammary gland, uptake by alveolar cells, and secretion in milk, would be required, and considerable amounts of pmiRNAs should be present in breast milk to significantly affect the infant [28,48]. To date, no direct quantitative evidence supports this cascade.

From an evolutionary perspective, the hypothesis of cross-kingdom gene regulation by dietary plant miRNAs in mammals raises several conceptual challenges. Plants and animals diverged more than 1.6 billion years ago and have since evolved distinct miRNA biogenesis pathways, effector complexes, and target-recognition rules [127]. In plants, miRNAs typically exhibit near-perfect complementarity with their targets and induce mRNA cleavage, whereas in animals, partial complementarity predominates, leading mainly to translational repression [15]. These fundamental differences suggest that plant miRNAs are unlikely to be evolutionarily optimized to regulate mammalian transcripts. Furthermore, no clear selective pressure supports the evolution of plant miRNAs that specifically target mammalian genes in a beneficial manner. From a fitness standpoint, it would be counterintuitive for plants to evolve regulatory molecules that enhance the physiology of herbivores or consumers, as plant–animal interactions are predominantly shaped by defensive strategies, including physical barriers and secondary metabolites [128].

An additional constraint is represented by the extremely low physiological concentrations at which dietary pmiRNAs have been detected in mammalian systems. Current quantitative estimates suggest that these molecules are present at femtomolar levels, several orders of magnitude lower than endogenous miRNAs known to exert regulatory effects. Such concentrations are unlikely to achieve sufficient stoichiometric interaction with target mRNAs to produce measurable gene silencing under physiological conditions [54].

Taken together, these considerations argue against a classical model of evolutionarily selected cross-kingdom gene regulation. However, they do not entirely exclude the possibility of indirect or context-dependent effects. For instance, pmiRNAs may influence host physiology through interactions with the gut microbiota, or they may represent incidental molecular interactions arising from sequence similarity rather than adaptive evolution [23,24,63]. In this framework, any observed cross-kingdom effects may reflect non-adaptive by-products rather than biologically selected regulatory mechanisms. Even weak or sparse molecular inputs may be amplified through non-linear dynamics or collective effects, giving rise to emergent phenotypic consequences that are not readily captured by reductionist models of miRNA–target interaction. Accordingly, while a classical paradigm of evolutionarily selected cross-kingdom gene regulation appears unlikely, the possibility of indirect, non-adaptive influence of dietary miRNAs on host physiology remains an open and potentially informative area of investigation.

From a methodological perspective, contamination remains a major confounding factor at all steps of pmiRNA analysis, from extraction to sequencing and qRT-PCR. Rigorous use of negative controls, spike-ins, and standardized reporting criteria is therefore critical to differentiate genuine exogenous signals from technical noise. As recently emphasized, the absence of widely accepted reference standards represents a key obstacle to reproducibility.

Although the debate is still open, demonstrating the ability of pmiRNAs to regulate gene expression in mammals would have far-reaching clinical, nutritional, and ecological implications. Indeed, the therapeutic potential of pmiRNAs is being investigated, in the attempt to better understand the mechanisms underlying traditional medicine and phytotherapy, as well as provide the foundations for novel plant-based therapies and nutrigenomic studies [129]. Furthermore, native or engineered plant-derived EVs are emerging as promising biocompatible nanocarriers for RNA-based drug delivery, allowing advances in this field directly relevant to therapeutic innovation [130]. Future research should adopt quantitative and methodologically rigorous frameworks, integrating in silico, in vitro, and in vivo approaches under contamination-controlled conditions. Particular attention should be given to (i) dose–response experiments at physiologically realistic levels, (ii) standardized controls and spike-ins, and (iii) functional validation through reporter assays.

Based on current methodological and biological evidence, cross-kingdom regulation by pmiRNAs remains a controversial and largely unproven hypothesis. Nevertheless, the exploration of how plant-derived RNAs interact with mammalian and microbial systems continues to challenge our understanding of gene-environment communication. Clarifying this phenomenon, whether to confirm or definitively refute it, will be critical for advancing both molecular medicine, and RNA biology.

## 6. Conclusions

Since L. Zhang et al. first proposed the hypothesis of dietary uptake of pmiRNA and cross-kingdom regulation in mammals, multiple studies have provided evidence supporting pmiRNA stability under the unfavorable conditions imposed by food processing, mastication, and digestion [21].

Plant-derived EVs have been suggested to contribute to pmiRNA stability in the GI tract and their uptake by intestinal and bacterial cells. Although evidence seems pointing in that direction, the emerging interest in RNA-based drug delivery using plant-derived EVs will certainly help deepen our knowledge on the topic. Moreover, pmiRNAs could modulate the mammalian microbiota through mechanisms similar to those well-established in plants. Though plausible, more evidence is certainly needed to corroborate it.

The most controversial question in the field is whether dietary pmiRNAs can cross the GI barrier and enter circulation in meaningful amounts to reach their targets and regulate their expression. Little is known on the mechanisms of pmiRNA transfer in bloodstream, as well as cellular uptake of circulating miRNAs. Importantly, contamination and sequencing artifacts have been shown to occur frequently, further mining the validity of evidence supporting cross-kingdom gene expression regulation by dietary pmiRNAs. Still, few functional studies indicate that pmiRNAs can regulate gene expression in mammalian cells, leaving the question open. Clarifying this phenomenon through quantitative and methodologically rigorous research will be critical for advancing both RNA biology and molecular medicine. Indeed, even if the transfer of pmiRNAs through physiologic, dietary intake was definitively refuted, their use as supplements and/or therapeutics via targeted delivery could still represent an interesting possibility.

## Figures and Tables

**Table 1:** Studies on pmiRNA stability during food processing and digestion.

Plant Species	Study Approach	Procedure	PmiRNAs Analyzed	Method	PmiRNA Recovery	Ref.
>Soybean (*Glycine max*)	In vitro	Soaking (ON); boiling (80 min); gastric digestion (pH 1.2, measured after 15, 30, 45, 60, 75 min)	miR-166; miR-167; miR-168	Stem-loop qRT-PCR	All pmiRNAs: ↑ soaking; ↑ boiling; ~ gastric digestion	[55]
Rice (*Oryza sativa*)		Boiling (25 min); gastric digestion (pH 1.2, measured after 15, 30, 45, 60, 75 min)			All pmiRNAs: ~ boiling; gastric digestion	
Corn (*Zea mays*)	In vitro	Boiling (20 min); drying (140 °C, 3 h; 65 °C, 36 h); puffing	miR-164a-5p; miR-319a-3p; miR-167e-5p/g-3p; miR-408a-3p; miR-166a-3p; miR-390a-5p; miR-159a-3p; miR-396a/c-5p; miR-168a-5p; miR-399a-3p; miR-171d-5p; miR-156a-5p; miR-2275b-5p; miR-162-5p; miR-827-3p/5p	sRNA-seq; Poly-A qRT-PCR	All pmiRNAs: ↓ boiling; drying; puffing	[32]
	In vivo	Pigs provided fresh maize diet (7 days). Heart, brain, lungs, kidney, mammary gland, muscle, liver, adipose tissue, spleen, pancreas collected at day 7		Poly-A and stem-loop qRT-PCR (w and w/o periodate oxidation)	miR396a-5p and miR827-5p: N.D. in any tissue Other pmiRNAs (w periodate oxidation): ↑↓	
		Pigs provided one fresh maize meal after ON fasting; fresh maize diet ad libitum (7 days). Serum collected at 0, 1, 3, 6, 12, 18, 24 h; 1, 3, 7 days	miR-164a-5p; miR-166a-3p; miR-167e-5p; miR-168a-5p; miR-319a-3p	Poly-A and stem-loop qRT-PCR	All pmiRNAs: ↑ 6–12 h after one meal: ~ after 7 days (~58.2% in EVs)	
	Ex vivo	Everted gut sac (measures at 0, 30, 60, 120 min)	Synthetic or fresh maize juice-derived miR-164a-5p; miR-167e-5p; miR-168a-5p	Poly-A and stem-loop qRT-PCR	All pmiRNAs: ↑	
Mistletoe (*Viscum album*)	In vitro	Drying; lyophilization; mechanical treatment; infusion (80 °C, 10 min); boiling (30 min)	miR-166a-3p; miR-159a; miR-831-5p; miR-218; miR-11	Stem-loop qRT-PCR	miR-166a-3p, miR-159a and miR-831-5p: ~ herbal preparations; N.D. after boiling Other pmiRNAs: ~ herbal preparations	[61]
Ginseng (*Panax ginseng*)	In vitro	Infusion (85 °C, 2 h)	miR-168; miR-156; miR-165; miR-394; miR-167; miR-162; miR-166; miR-159; miR-396	sRNA-seq; Poly-A qRT-PCR	miR-168 and miR-156: ~ Other pmiRNAs: ↓	[62]
N.A.	In vitro	Gastric digestion (pH 2.5, measures at 0, 10, 30, 60, 90, 120 min, w or w/o food macronutrients); intestinal digestion (pH 7.8, measures at 0, 30, 60, 90, 120, 150, 180 min, w or w/o food macronutrients)	Synthetic (w or w/o 2′-O-Me, or FITC-modified) ahy-miR-157a; sly-miR-172a; sbi-miR-894; bol-miR-159; gma-miR-160; osa-miR-168a	Poly-A qRT-PCR	All pmiRNAs: ↓↓ digestion phases; ↓ gastric digestion w food macronutrients; ↓ intestinal digestion w 2′-O-Me	[56]
Broccoli (*Brassica oleracea* var. *italica)*	In vitro	Sprouting, juicing (4 min in 60 mL water); boiling (15 min); steaming (15 min); frying (8 min); microwaving (2 min); blanching (2 min); salivary digestion (pH 7.0, 2 min); gastric digestion (pH 2.5, 1 h); intestinal digestion (pH 7.0, 2 h)	miR-165a-5p; miR-168a; miR-167a/b/c; miR-165b; miR-166a/b/c/d/e	Poly-A qRT-PCR	All pmiRNAs: ↑↓ food processing; miR-165a-5p: ~ digestion phases Other pmiRNAs: ~ salivary and gastric digestion; ↓ intestinal digestion	[57]
N.A.	In vitro	Boiling (5 min or 1 h); gastric digestion (pH 2, 1 h); intestinal digestion (pH 7.0, 1 h); pH 12 treatment; RNase A treatment	Synthetic ath-miR-171a-3p	Poly-A qRT-PCR	N.D. after pH 12 and RNase A treatment; ~ digestion phases; ~ boiling	[58]
N.A.	In vitro	Salivary digestion (measures at 0, 2, 4, 6 min, w or w/o food macronutrients)	Synthetic and native (w or w/o 2′-O-Me) ahy-miR157a; sly-miR-172a; sbi-miR-894; bol-miR-159; gma-miR-160; osa-miR-168a	Stem-loop qRT-PCR	All pmiRNAs: ↓↓ salivary digestion; ↓ salivary digestion w food macronutrients; ↓ salivary digestion w 2′-O-Me	[60]
Orange (*Citrus x sinensis*)			EV-contained miR-482a; miR-166e; miR-390a; miR-159; miR-166b		miR-482a and miR-166a: ↓ salivary digestion; ~ salivary digestion w food macronutrients Other pmiRNAs: ~ digestion conditions	
N.S.	In vivo	Human subjects followed enriched plant-based diet (3 days) after a wash-out period (poor plant intake, 2 days). Serum and feces collected before and after the intervention	miR-156e; miR-159; miR-162	Poly-A qRT-PCR	All pmiRNAs: N.D in serum miR-156e and miR-162: ↑ in feces miR-159: ~ in feces	[63]

ON = overnight; N.D. = not detected; N.A. = not applicable; N.S. = not specified, ↑ = highly expressed, ↓ = lowly expressed.

**Table 2:** Best practices and recommended controls for pmiRNA studies (design → detection → interpretation).

Workflow Step	Potential Bias/Risk	Recommended Best Practice (Actionable)	Controls to Include	Reporting Minima
Study design and preregistration	Underpowered Selective reporting	Perform power analysis Preregister analysis plan Define a priori primary endpoints (presence, absolute copies, effect)	Independent biological replicates Randomized batches	Sample size justification Randomization and blinding
Dietary exposure definition	Non-physiological doses Uncertain intake	Use nutritionally relevant doses Record diet logs Replicate real prep (boiling/steaming)	Placebo/control diet Dose–response arms	Exact mass/serving Prep method Timing versus sampling
Sample collection	Environmental carry-over Hemolysis/lipemia artifacts	Utilize dedicated clean areas Use RNase/DNase-free plasticware and reagents Change gloves/tips frequently	Air/bench swabs Field blanks	Time-to-freeze Hemolysis index Storage temp/time
Extraction	Kit-borne sRNA contaminants Variable yield	Prefer phenol–chloroform for low abundance targets Maximize input Co-extract process controls	Extraction blanks (no input) Spike-ins added before lysis	Method/kit lot Input volume/mass Recovery yield
Contamination monitoring (global)	Cross-sample/library carry-over	Follow unidirectional workflow (physically separate pre/post-PCR steps) Use filter tips	No-template controls (NTC) at RT and PCR Library blanks	Frequency of positives in blanks Any remediation
Library prep—adapter ligation	Under-representation of pmiRNAs (2′-O-Me ligation bias)	Optimize ligation: longer incubation, ↑ ligase, PEG (crowding) Use randomized adapters Utilize unique molecular identifiers (UMIs)	Parallel library with optimized ligation Ligation-only negative	Enzyme Incubation times PEG % Adapter sequences
Plant vs. animal miRNA discrimination	Mis-assignment/cross-mapping	Perform periodate oxidation prior to library prep (enriches 2′-O-Me pmiRNAs) Carry out stringent multi-genome mapping with seed-mismatch filters	Oxidized vs. non-oxidized library pair Shuffled-seed control	Mapper Software versions Reference builds Mismatch policy
Periodate oxidation and β-elimination	Insufficient input Partial oxidation	Validate oxidation on controls Scale input Include oxidation efficiency check	Synthetic methylated and unmethylated spike-ins	Input mass Oxidation efficiency read-outs Batch variability
Sequencing depth and batch	Low copies miss due to low depth Batch effects	Sequence at depth ≥ 10–20 M reads/library for discovery Randomize across lanes Use dual indexing strategies	Technical replicates across lanes Batch calibrators	Exact depth Lane allocation Index set
Bioinformatics	Index hopping Pipeline artifacts	Remove exact duplicates via UMIs Filter low-complexity sequences Perform joint decontamination with extraction and library blanks	Prevalence-based methods (e.g., Decontam) Cross-study pattern check	Full pipeline Software versions Parameters Publicly available FASTQ files
qRT-PCR chemistry	Under-detection of 2′-O-Me pmiRNAs by poly(A)-tailing	Prefer stem-loop qRT-PCR Use locked nucleic acid (LNA) probes if needed	RT-minus NTC Standard curves per target	Primer/probe sequences Efficiency (90–110%) R^2^
Mature vs. immature pmiRNA discrimination	Immature/unmethylated pmiRNA amplification	Perform periodate oxidation and β-elimination prior to qRT-PCR to select mature 2′-O-Me pmiRNAs	Synthetic methylated and unmethylated spike-ins	ΔCt (treated vs. untreated) per target
Spike-ins and normalization	Non-comparable concentrations Drift	Add synthetic 2′-O-Me spike-ins at fmol-pmol levels (comparable to input miRNAs) pre-extraction Use ≥2 spike-ins Perform inter-plate calibration	Standard curves per target Process-matched spike-ins added before lysis	Absolute copy number (copies/mL or copies/µg RNA) Spike-in identity and input amount Calibration model used
Endogenous controls	Unstable reference in fluids	Validate candidate references (genorm/normfinder) Avoid hemolysis-sensitive miRNAs	Panel of candidate references Stability report	Stability metrics Chosen normalizer rationale
EV isolation and attribution	Precipitation carry-over Non-EV complexes	Prefer differential ultracentrifugation (UC)/size-exclusion chromatography (SEC) and density gradient UC Characterize particles following MISEV guidelines (size, markers)	Protease + RNase ± detergent protection assays	EV yield and size by Nanoparticle Tracking Analysis (NTA) Markers (CD63/Alix) Purity Protection index
Evidence of encapsulation	Protein/RNP protection misread as EV encapsulation	Perform buoyant density profiling Compare AGO pull-down vs. EV fractionation	RNase protection assay RNase ± detergent ± protease triage Fraction-specific qPCR	Protection index (RNase vs. RNase + detergent) EV purity metrics (NTA, markers)
Functional validation	Correlation ≠ causation	Dual-luciferase reporter with human 3′UTR Site mutagenesis Dose–response at physiological fmol-pmol levels	Mutant seed controls Scrambled pmiRNAs	EC50/IC50 Effect size at physiological doses
In vivo plausibility	Unrealistic dosing Confounders	Adopt an effective PK/PD sampling strategy (0–24 h) Perform mass balance studies Prescribe realistic diets Set blinded endpoints	Vehicle diet Pair-feeding Cross-over where feasible	Cmax/Tmax Absolute copies in plasma/tissue
Microbiota pathway	Host vs. lumen conflation	Collect feces and plasma in parallel Carry out 16S/shotgun + metabolomics Use antibiotic/depletion models	Blood-negative/feces-positive pattern as criterion	Microbiota metrics Targeted metabolites
Data transparency	Non-reproducibility	Deposit raw FASTQ, Ct tables, calibration curves, full methods	Public accession Analysis notebooks	Accession IDs Reagent lots

## Data Availability

No new data were created or analyzed in this study. Data sharing is not applicable to this article.

## Abbreviations

The following abbreviations are used in this manuscript:

miRNAs	micro-RNAs
pmiRNAs	plant-derived microRNAs
EVs	Extracellular vesicles
GI	Gastrointestinal
LDLRAP1	Low-density lipoprotein receptor adapter protein 1
ON	Overnight
N.D.	Not detected
N.A.	Not applicable
N.S.	Not specified
2′-O-Me	2′-O-methylation
SIDT1	SID-1 transmembrane family member 1
sRNA-seq	Small RNA-sequencing
BC	Breast cancer
CVD	Cardiovascular disease
JAM-A	Junction adhesion molecule-A
PCR	Polymerase chain reaction
qRT-PCR	Quantitative reverse transcriptase-PCR
PEG	Polyethylene glycol
